# Nature of novel criticality in ternary transition-metal oxides

**DOI:** 10.1038/s41598-019-55594-w

**Published:** 2019-12-18

**Authors:** Shapiullah B. Abdulvagidov, Shamil Z. Djabrailov, Belal Sh. Abdulvagidov

**Affiliations:** 1 Institute of Physics, Dagestan Science Centre of RAS, 94, Makhachkala, 367003 Russian Federation; 2 Hadzhalmakhi secondary school, Hadzhalmakhi, 368317 Russian Federation; 3 Dagestan State University, Makhachkala, 367000 Russian Federation

**Keywords:** Electronic properties and materials, Ferromagnetism, Magnetic properties and materials, Phase transitions and critical phenomena, Spintronics

## Abstract

There are the chains of transition-metal cations alternating with the anions of oxygen in ternary transition-metal oxides. When a *p*-orbital of the oxygen connects the half-filled and empty *d*-orbitals of adjacent transition-metal cations, double-exchange ferromagnetism takes place. Although double exchange has been well explored, the nature of novel criticality, induced by it, is yet not uncovered. We explored the magnetic-field scaling in the heat capacity of a Sm_0.55_Sr_0.45_MnO_3_ manganite, one of the best ternary transition-metal oxides as it is completely ferromagnetic, and found novel criticality - unordinary critical exponents which are the consequence of coherence of Coulomb lattice distortion and ferromagnetism. The coherence is caused by the trinity of the mass, the charge and the spin of an electron. When the *d* and *p* orbitals overlaps, it quickly walks from one site to the another due its lightest mass. And due to its electric charge, it equalizes the valences of the transition-metal cations in the chains and so diminishes the Coulomb lattice distortion. At last, its spin forces magnetic moments of transition-metal cations to ferromagnetically arrange. The disappearance of Coulomb distortions widens the overlap and lowers the elastic lattice energy, so that not only the spin of an electron, but also its electric charge strengthens ferromagnetism. That nonlinear effect strengthens the critical behaviour and critical exponents come off any known universality classes. Thus, the symbiotic coherence of annihilating Coulomb distortions and arising ferromagnetism is a reason of the novel criticality.

## Introduction

Critical phenomena at a second-order phase transition provides valuable information about a solid, such as the symmetry, dimensionality and energy of ordering interaction, the size of the droplets of a nascent phase and the type of disorder - static, dynamical, frozen-in, or annealed, and even are very fruitful at exploring cosmological problems^1^. Nevertheless, the nature of the novel criticality, critical exponents out of any known universality classes, near Curie point in ternary transition metal oxides (TTMO) - manganites, cobaltites, nickelates, and etc. - keeps unknown^2–10^. During half a century, TTMO are one of the foci for condensed-matter physics, not only opening pathways for applied physics to fabricate new spintronic devices, but also giving an impulse for the development of pure physics. Despite the huge number of publications, dealt with the scaling of the magnetization and susceptibility in TTMO, however, there is only single attempt^11^ of the heat-capacity scaling in La_0.7_Ca_0.3_MnO_3_ manganite, which has not shown the collapse of the heat capacity in La_0.7_Ca_0.3_MnO_3_ manganite into a single scaling function, because of, unlike an ordinary ferromagnet, the heat-capacity peak shifted substantially in temperature. An improved scaling procedure taking into account the strong sensitivity of *T*_C_ to magnetic field and hysteresis allowed us to reach the collapse of the magnetic heat capacity in La_0.85_Ag_0.15_MnO_3_ manganite^12^. Not having more literature data upon the scaling of heat capacity, below we juxtapose through scaling equalities the critical exponents of the heat capacity α and correlation radius ν with the magnetic critical exponents - spontaneous magnetization *β*, magnetic susceptibility *γ*, and isothermal magnetization *δ*.

Unordinary critical behaviour is observed in qualitatively various compounds, for example, in TTMO, in a 3*d*-metal alloys^9^, in a fullerene^2^, and in a ferromagnetic uranium superconductor^8^. Nevertheless, they are united by the common nature of their novel ferromagnetic criticality accompanied with the lattice distortions. Griffiths phase, a feature of mesoscopic lattice and magnetic inhomogeneities, was observed to cause unusual critical exponents *β*, *γ*, and *δ* in La_0.79_Ca_0.21_MnO_3_, whereas La_0.80_Ca_0.20_MnO_3_ free of the Griffiths phase belongs to Heisenberg model (Table 1, rows 13 and 20). The magnetization in iron-doped La_0.7_Ca_0.3_Mn_1−x_Fe_x_O_3_ manganites reveals two unknown universality classes below and above *T*_C_, because of the ferromagnetic interaction has disordered and itinerant character by increasing Fe concentration, although the *β* and *γ* obeys scaling relations^10^. It is obvious that the substitution of Mn by Fe, breaking Mn-O-Mn chains, induces the lattices distortions and disorders the double-exchange ferromagnetism, and so the critical phenomena in La_0.7_Ca_0.3_Mn_1−x_Fe_x_O_3_ could not be explained on the conventional universality classes. Also two critical behaviours take place in an manganese nitride Cu_0.9_NMn_3.1_: ordinary mean field theory below *T*_C_, but above *T*_C_ robust critical fluctuations with (*β*, *γ*) = (0.532, 1.63) out of any universal classes (it should be noted that these values lead to unusually large negative *α* = 2(1 − *β*) − *γ* = −0.694 out of any universal classes too), which explained by the short-range antiferromagnetic ordering above *T*_C_^9^. Critical exponents *β*, *γ*, *δ* in an organic ferromagnetic tetrakis(dimethylamino)ethylene fullerene[60] differ significantly from the 3D-Heisenberg and, in addition, do not obey the scaling *γ* = *β*(*δ* − 1) and superscaling *γ* + 2*β* = dν relations^2^, that are attributed to the presence of disorder in the C_60_ molecular orientations near *T*_C_. The magnetization of UGe_2_ and URhGe shows strong uniaxial magnetic anisotropy, nevertheless, their universality class is no 3D-Ising and cannot be explained via previous approaches to the critical phenomena^8^. It is obvious that the unconventional criticality is also caused by lattice distortions with appearing the strong itinerant character of the 5*f* electrons in the ferromagnetic superconductive state. Thus, not only the percolation of the 3*d* electrons but also the delocalization of the 5*f* electrons can lead to a novel criticality.

**Table 1:** Critical parameters of representatives of the TTMO universality class with members of the known universality classes reported in literature.

Rows	Model or compound	β	γ	δ		α		ν^e^	Refs
**1**	**Mean field model**	**0,50**	**1**	**3,00**	**3,00**^**a**^	**0,00**		**0,67**	^20,39^
2	La_0.75_Sr_0.25_CoO_3_	0,36	1,30	4,75	4,60^a^	−0,03^d^	−0,08^c^	0,68	^39^
3	La_0.67_Sr_0.33_CoO_3_	0,36	1,31	4,61	4,63^a^	−0,03^d^	−0,03^c^	0.68	^40^
4	La_0.7_Sr_0.3_Mn_0.97_Ni_0.03_O_3_	0,468	1,010	2,67	3,16^a^	0,05^d^	0,28^c^	0,65	^39^
5	La_0.7_Ca_0.3_Mn_0.91_Fe_0.09_O_3_	0,42	1,20	3,70	3,84^a^	−0,05^d^	0,01^c^	0,68	^10^
6	La_0.7_Ca_0.3_Mn_0.89_Fe_0.11_O_3_	0,46	1,06	3,36	3,31^a^	0,01^d^	−0,01^c^	0,66	
7	Cu_0.6_NMn_3.4_	0,481	1,09	3,22	3,27^a^	−0,05^d^	−0,03^c^	0,68	^9^
8	(V_0.989_Cr_0.011_)_2_O_3_	0,50	1,00	3,00	3,00^a^	0,00^d^	0,00^c^	0,67	^3^
**9**	**3D Heisenberg Model**	**0,365**	**1,386**	**4,80**	**4,80**^**a**^	**−0,12**		**0,71**	^20,39^
10	Amorphous Fe_80_P_13_C_7_	0,38	1,30	4,47	4,42^a^	−0,06		0,69	^41^
11	Nickel	0,38	1,34	4,58	4,54^a^	−0,10		0,70	^42^
12	BaRuO_3_	0,348	1,410					0,70	^6^
13	La_0.80_Ca_0.20_MnO_3_	0,365	1,369	4,8	4,75^a^	−0,10^d^		0,70	^5^
14	SrFe_0.80_Co_0.20_O_3.0_	0,390	1,360					0,71	^27^
15	Amorphous Gd_80_Au_20_	0,44	1,29	3,96	3,93^a^	−0,17		0,72	^43^
**16**	**3D Ising Model**	0,325	1,241	4,82	**4,82**^**a**^	**0,11**		0,63	^20,39^
17	Gadolinium	0,38	1,19	3,61	4,13^a^	0,06		0,65	^44^
18	La_0.66_Pb_0.34_MnO_3_	0,24	1,464	7,1	7,10^a^	0,06^d^	0,06^c^	0,65	^45^
19	La_0.7_Sr_0.3_MnO_3_	0,31	1,271^b^	5,1	5,10^a^	0,11^d^	0,11^c^	0,63	
20	La_0.79_Ca_0.21_MnO_3_	0,09	1,71	20,0	20,00^a^	0,11^d^	0,11^c^	0,63	^46^
21	La_0.7_Sr_0.3_Mn_0.99_Ni_0.01_O_3_	0,394	1,092	3,99	3,77^a^	0,12^d^	0,03^c^	0,63	^39^
22	La_0.7_Sr_0.3_Mn_0.98_Ni_0.02_O_3_	0,400	1,018	3,79	3,55^a^	0,18^d^	0,08^c^	0,61	
**23**	**3D-XY**	**0,34**	**1,30**	**4,82**	**4,82**^**a**^	**−0,014**	**0,02**^**c**^	**0,66**	^19^
24	(Sm_0.7_Nd_0.3_)_0.52_Sr_0.48_MnO_3_	0,358	1,297	4,536	4,62^a^	−0,01^d^	0,02^c^	0,67	^6^
25	Iron	0,37	1,30			−0,04		0,68	^47^
**26**	**Chiral Heisenberg**	**0,30**	**1,17**	**4,90**	**4,90**^**a**^	**0,240**		**0,59**	^19^
27	La_0.7_Ca_0.3_MnO_3_	0,10	1,590^b^	16,90	16,90^a^	0,21^d^	0,21^c^	0,60	^45^
**28**	**Chiral XY**	**0,25**	**1,13**	**5,52**	**5,52**^**a**^	**0,340**	**0,37**^**c**^	**0,54**	^19^
29	La_0.9_Te_0.1_MnO_3_	0,20	1,27	7,14	7,32^a^	0,33^d^	0,36^c^	0,56	^48^
30	La_0.1_Nd_0.6_Sr_0.3_MnO_3_	0,26	1,12	5,17	5,36^a^	0,37^d^	0,41^c^	0,54	^49^
31	UGe_2_	0,329	1,02			0,32^d^		0,56	^8^
32	URhGe	0,302	1,02			0,38^d^		0,54	
**33**	**Tricritical mean-field theory**	**0,25**	**1,0**	**5,0**	**5,0**	**0,50**		**0,5**	^50^
34	C_6_Li	0,22				0,50		0,15	^51^
35	La_0.7_Ca_0.3_MnO_3_	0,25	1,0			0,50^d^		0,50	^46^
**36**	**TTMO**	**0**,**45**	**1**,**36**	**4**,**14**	**4**,**03**^**a**^	**−0**,**25**	**−0**,**28**	0,75	This work
37	Fe_70_Pt_30_	0,46	1,28	3,80	3,78^a^	−0,20^d^	−0,21^c^	0,73	^52^
38	(Fe_0.68_Mn_0. 32_)_75_P_16_B_6_Al_3_	0,40	1,40^b^	4,50	4,50^a^	−0,20^d^	−0,20^c^	0,73	^53^
39	YBa_2_Cu_3_O_7−δ_					−0,25		0,75	^54^
40	La_0.7_Ba_0.3_MnO_3_	0,35	1,41	5,50	5,03^a^	−0,28^d^	−0,28^c^	0,70	^55^
41	La_0.7_Sr_0.3_CoO_3_	0,43	1,43	4,38	4,33^a^	−0,29^d^	−0,31^c^	0,76	^56^
42	La_0.79_Sr_0.21_CoO_3_	0,49	1,22	3,51	3,48^a^	−0,20^d^	−0,21^c^	0,73	^45^
43	La_0.85_Ag_0.15_MnO_3_					−0,23		0,7433	^12^
44	Sm_0.55_Sr_0.45_MnO_3_					−0,23		0,7433	This work
45	Fe_72_Pt_28_	0,49	1,36	3,70	3,78^a^	−0,34^d^	−0,30^c^	0,78	^52^
46	Fe_70_Pt_30_	0,49	1,33	3,80	3,71^a^	−0,31^d^	−0,35^c^	0.77	
47	La_0.80_Sr_0.20_CoO_3_	0,46	1,39	4,02	4,02^a^	−0,31^d^	−0,31^c^	0,77	^56^
48	La_0.75_Sr_0.25_CoO_4_	0,46	1,39	4,02	4,02^a^	−0.31^d^	−0,31^c^	0,77	
**49**	**“Random fixed point”**	**0**,**5**	**2**,**0**	**5**,**0**	**5**,**00**^**a**^	**−1**,**0**		**1.0**	^52^
50	Fe_75_Pt_25_	0,50	1,62	4,20	4,24^a^	−0,62^d^	−0,60^c^	0,87	^52^
51	Fe_74_Pt_26_	0,48	1,68	4,40	4,54^a^	−0,63^d^	−0,57^c^	0,88	
52	Fe_74_Pt_26_	0,50	1,75	4,40	4,50^a^	−0,75^d^	−0,70^c^	0,92	
53	Fe_74_Pt_26_	0,49	1,90	5,00	4,88^a^	−0,88^d^	−0,94^c^	0,96	
54	Fe_70_Pt_30_	0,50	1,60	4,15	4,20^a^	−0,60^d^	−0,58^c^	0,87	
55	(Fe_0.68_Mn_0. 32_)_75_P_16_B_6_Al_4_	0,40	1,67^b^	5,00	5,00^a^	−0,40^d^	−0,40^c^	0,80	^53^
56	Metglass 2826A (Fe_32_Ni_36_Cr_14_P_12_B_6_)	0,41	1,67	5,07	5,07^a^	−0,49	−0,49	0,83	^57^
57	Fe_75_Pt_25_	0,50	1,42	3,80	3,84^a^	−0,42^d^	−0,40^c^	0,81	^52^
58	Cu_0.7_NMn_3.3_	0,5	1,31	3,57	3,40^a^	−0,40^d^	−0,50^c^	0,80	^9^
59	Cu_0.9_NMn_3.1_	0,5	1,63	3,84	4,06^a^	−0,69^d^	−0,57^c^	0,90	
60	Fe_72_Pt_28_	0,50	1,49	3,95	4,01^a^	−0,48^d^	−0,45^c^	0,83	^52^
61	TDAE-C_60_	0,75	1,22	2,28	2,63^a^	−0,72^d^	−0,46^c^	0,91	^2^
62	BaIrO_3_	0,82	1,03	2,20	2,26^a^	−0,67^d^	−0,62^c^	0,89	^58^
63	κ-(BEDT-TTF)_2_X	1,00	1,00	2,00	2,00^a^	−1,00^d^	−1,00^c^	1,00	^4^

In the rows 52, 53, 58, 59 and 61, compounds violate the scaling equalities with isothermal *δ*. In the rows 4–6, 21, 22 and 24, compounds violate scaling equalities because of desynchronizing their lattice and magnetic behaviours. Although measured *β*, *γ* and *δ* in some compounds say about the novel criticality, their *α* and *v* calculated via scaling relations indicate an ordinary universality class (rows 61–63). “A value with ^**a**^Сalculated from Widom scaling” equality δ = 1 + γ/β, with ^**b**^From Widom scaling equality γ = β(δ − 1), with ^c^From combining Widom and Rushbrook scaling equalities α = 2 − β(1 + zδ), with ^**d**^From Rushbrook scaling equality α = 2(1 − β) − γ, and with ^**e**^From Josephson superscaling equality dν = γ + 2β at d = 3.

Pressure^3,4,6,7^, modifying critical behavior by way of sizably reducing the lattice spacing, hints at a key role of lattice distortions in novel criticality. Pressure^6^ enhances critical ferromagnetic behaviour in BaRuO_3_, whereas keeps mean-field behavior in SrRuO_3_. The application of pressure^7^ to (Sm_0.7_Nd_0.3_)_0.52_Sr_0.48_MnO_3_ single crystal increases the *T*_C_ and suppresses the hysteresis that makes the transition a second order with (*β*, *γ*, *δ*) = (0.358, 1.297, 4.536) close to the 3D-Heisenberg at 12.1 kbar. The quasi-two dimensional organic insulator κ-(BEDT-TTF)_2_Cu[N(CN)_2_]Cl shows a pressure-induced critical behaviour inconsistent with any universal classes, hence novel criticality is inherent in quasi-two dimensional systems, in which correlated electrons form a many-body system with anomalous collective behaviour, which cannot be understood from known spin models and, therefore, it needs some new explanation for the unconventional exponents^4^ (*β*, *γ*, *δ*) ≈ (1, 1, 2).

It should be noted that double exchange is qualitatively different from ordinary exchanges – direct exchange, *s*-*d*-, *s*-*f*- and *RKKY*-exchanges, and superexchange; as it can alter critical properties and even introduce new universality classes^13^, lattice distortions and “metallization” being co-occurred. Whereas direct exchange, *s*-*d*-, *s*-*f*- and *RKKY*-exchanges, and superexchange do not qualitatively change the electrical behavior. A ferromagnetic with direct exchange, *s*-*d*-, *s*-*f*- and/or *RKKY*-exchanges is metallic below and above Curie point. And an antiferromagnetic with *RKKY*-exchange or superexchange is insulating or semiconducting below and above Neel point. In contrast to the ordinary ferromagnetic exchanges antagonistic to superexchange, the double exchange mutually coexists with the superexchange: ‘temperature – doping level’ phase diagrams of TTMO shows adjacent ferromagnetic and antiferromagnetic areas, where the Neel point gradually transits into the Curie point and reverse. Superexchange, at which a *p*-electron of an anion stays in the *d*-orbitals of the adjacent Mn^2+^-cations only about 2% of the time, is virtual, as the electron must come back. That small time is not able to cause lattice distortions as the ions valent states below (Mn^1.98+^-O^1.98−^Mn^1.98+^ in MnO or Mn^2.98+^-O^1.98−^Mn^2.98+^ in an undoped manganite) and above (Mn^2+^-O^2−^Mn^2+^ or Mn^3+^-O^2−^Mn^3+^) Neel point are practically same and charge symmetry relative to an anion remains. Magnetics with *s*-*d*, *s*-*f*, or *RKKY* exchange also do not reveal sufficient lattice striction, as their *d* or *f* electrons do not walk between atoms but only polarize conduction electrons. Overlapping *d*-orbitals in ferromagnetic with direct exchange is less than 1% (the overlapping more 1% leads to antiferromagnetism) that makes it impossible colossal spontaneous striction. Double exchange involves actual electron hopping, as opposed to virtual electron hopping in the superexchange scenario: one electron from a Mn^3+^-cation transfers through intervened O^2−^anion into the adjacent Mn^4+^-cation and can either go back or go on to the next Mn^4+^-cation. In electric and/or magnetic fields, the onward motion prevails and electric current arises, which can switch magnetic structures and spintronic devices^14–18^. In contrast to all other kinds of exchange practically weakly influencing on lattice constants, double exchange, turning a mixed-valent system (Mn^3+^-O^2−^Mn^4+^) into the fractional valent one (Mn^3.5+^-O^2−^Mn^3.5+^), vanishes the mixed-valent-induced lattice distortions, the colossal spontaneous striction occuring. That striction strengthens the overlapping of the *d*- and *p*-orbitals that increases the integral of double exchange. Thus, double exchange not only gives birth to the double-exchange ferromagnetism but also governs its exchange integral through Coulomb distortion. That property of double exchange is unique. Then, in a double-exchange magnet, ferromagnetism grows near the Curie point much more quickly than in a canonical ferromagnet, that is, with *d*-*d*, *s*-*d*, *s*-*f*, and/or *RKKY* exchanges, and that TTMO manifest novel criticality must not be a surprise. The peculiarities of double exchange, mentioned here, allow us to consider it responsible for the novel criticality in a solid. Remarkable, binary transition-metal oxides, being antifferomagnetic, stay within the conventional criticality: ReMnO_3_ manganites (Re is a rare earth) belong either to the 3D-Heisenberg or to the 3D-XY in the critical exponents and amplitudes of the magnetization and heat capacity near Neel point^19^; The electrical conductivity of the antiferromagnetic (V_1−x_Cr_x_)_2_O_3_ Mott insulators reveals the mean field which goes into 3D-Ising at the critical pressure^3^, i.e., even pressure cannot take them out of an ordinary critical behaviour.

Having made a survey of the above-mentioned substances with unordinary criticality, we found their one common pattern - their lattice structures had the adjacent cation-anion elements. When the cation objects, separated by the anion unit, have the different valences, numbers of holes, and/or occupations of orbitals (for example, in manganites, Mn^3+^ and Mn^4+^ spaced by O^2−^), double exchange creates ferromagnetism and eliminates Coulomb distortions. Together both these processes increase the magnetic interaction range and the magnetic order parameter much quicker than in canonical magnetics, and the novel criticality induced. For a instance, in the k-(BEDT-TTF)_2_Cu[N(CN)_2_]Cl organic insulator, the cation unit is a BEDT-TTF dimer with one hole (i.e., the band is half-filled) and an anion element an insulating anion layer. The half-filled orbitals indirectly (via the anion complex) hybridizing, the Pauli exclusion principle forbids the double-exchange. That is why, the antiferromagnetic insulator is only allowable in the k-(BEDT-TTF)_2_Cu[N(CN)_2_]Cl instead of the ferromagnetic conductor.

In the Report, having carried out the scaling in the magnetic heat capacity of the Sm_0.55_Sr_0.45_MnO_3_ manganite, one of the best TTMO as it is completely ferromagnetic, and having compared our and literature data, we could understand that the nature of the novel criticality in TTMO is caused by the symbiotic coherence of annihilating Coulomb distortions and arising ferromagnetism, which both are triggered by double exchange, the double charge exchange vanishing Coulomb distortions and the double spin one creating ferromagnetism.

## Results and Discussion

**Figure 1:**
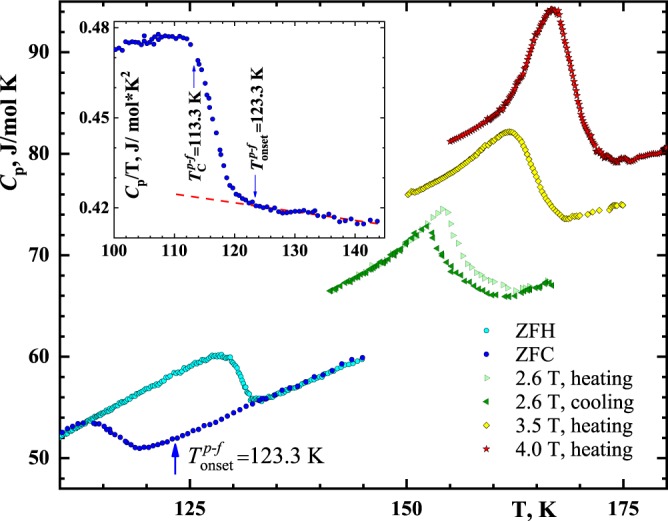
The temperature dependences of the Sm_0.55_Sr_0.45_MnO_3_ specific heat in the neighbourhood of the Curie points in the fields 0, 2.6, 3.5 and 4 T; there is no hysteresis at the two latter fields 3.5 and 4.0 T, so that the respective cooling and heating scans coincide. To avoid of superimposing, the graphs in the fields of 3.5 and 4 T are lifted upon 6.0 and 9.0 J/mol K. The insert shows how the temperature of the onset of ferromagnetism, ${T}_{onset}^{p-f}$, has been determined: A *C*_p_/T representation is very geometrically sensitive to fix ${T}_{onset}^{p-f}$, a temperature where heat capacity, because of appearing ferromagnetism, starts to inflect from the approximating straight line.

**Figure 2:**
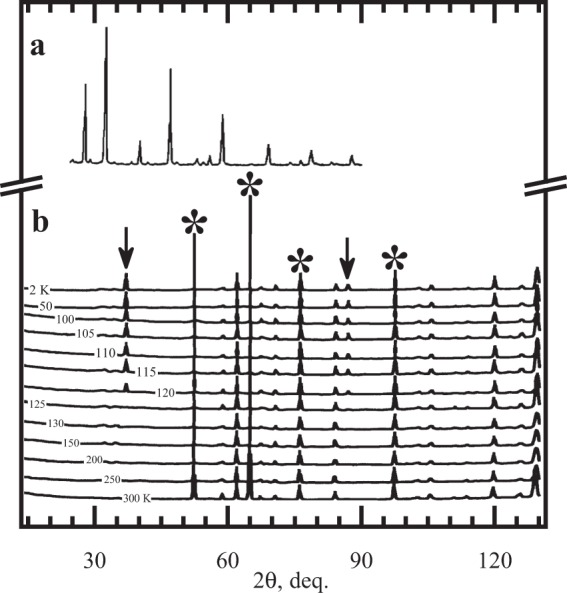
(**a**) The room-temperature XRD-patterns of ^152^Sm_0.55_Sr_0.45_MnO_3_. (**b**) The ZFC neutron-diffraction patterns of ^152^Sm_0.55_Sr_0.45_MnO_3_ at different temperatures. Arrows mark the first reflections attributed to bulk ferromagnetism. The first reflections attributed to metamagnetism are indicated with asterisks. The features of antiferromagnetism are absent. The 120-K scan is a first indication of ferromagnetism at temperature below the heat-capacity ${T}_{onset}^{p-f}$ = 123.3 K (see Fig. 1). The scans at the temperatures above ${T}_{onset}^{p-f}$ have no features of the ferromagnetic transition. Hence, not ${T}_{{\rm{C}}}^{p-f}$ but it is ${T}_{onset}^{p-f}$ which demonstrates itself at structural studies.

To prove our concept of a new universality class, the magnetic heat-capacity scaling of Sm_0.55_Sr_0.45_MnO_3_ manganite has been carried out. The zero-field and 2.6-T heat capacity in Sm_0.55_Sr_0.45_MnO_3_ display a Δ-shaped anomaly and hysteresis reflecting the first-order-like nature of the transition (Fig. 1). The zero-field cooling (ZFC) Curie point, that is, the paramagnetic-to- ferromagnetic transition (*p*-*f*), ${T}_{C}^{p-f}({\rm{ZFC}})$ = 113.3 K. The zero-field heating (ZFH) Curie point, the ferromagnetic-to-paramagnetic transition (*f*-*p*), ${T}_{C}^{f-p}({\rm{ZFH}})$ = 128.6 K. The ZFC inflection point at 123.3 K (Fig. 1, the insert), meaning the onset of ferromagnetism, is consistent with the 120-K neitron difraction scan (Fig. 2). Magnetic field, heightening ${T}_{C}^{p-f}$ faster than ${T}_{C}^{f-p}$, narrows their difference, i.e., the hysteresis. Hence, magnetic field suppresses hysteresis and completely vanishes it at 3.5 T and above, the Δ-shaped heat-capacity peak turning into λ-like. Hence, Sm_0.55_Sr_0.45_MnO_3_ behaves like an unordinary ferromagnet. That is because the intensification of fluctuations by the field-induced annihilating Coulomb distortion outweighs classic suppressing fluctuations by a magnetic field.

**Figure 3:**
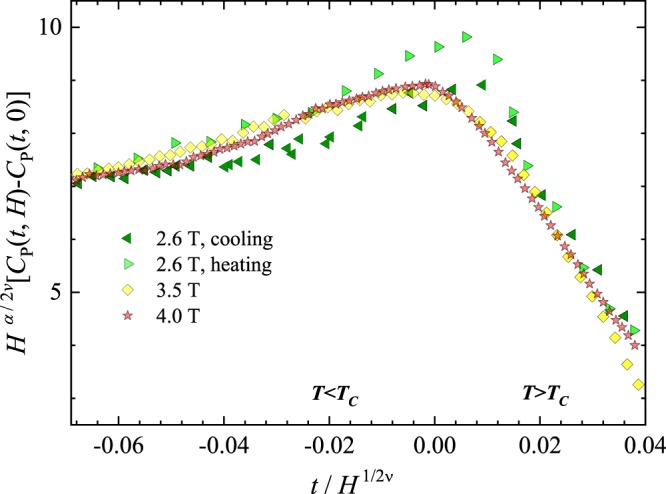
Collapse of the graphs *H*^*α*/*2*^ν [*C*_*p*_(*t*, *H*) − *C*_*p*_(*t*, 0)] vs. *t*/*H*^1*/2*^ν at (*α*, ν) = (−0.23, 0.7433) in Sm_0.55_Sr_0.45_MnO_3_ in the fields 2.6 T under cooling and heating, 3.5 T and 4.0 T.

The magnetic heat capacity in Sm_0.55_Sr_0.45_MnO_3_ was scaled according to the improved scaling procedure^12^. As Sm_0.55_Sr_0.45_MnO_3_ is hysteretic and high-sensitive to magnetic field, we took distinct values of *Т*_C_ for cooling and warming runs, and for each of magnetic fields and calculated the reduced temperature *t* = *T*/*T*_C_ − 1 and the magnetic heat capacity *C*_p_(*t*, *H*) − *C*_p_(*t*, 0) – the difference of heat capacities in a given field and without a field. If at a certain *α* and ν the scaling exists, dependences *C*_p_(*t*, *H*) − *C*_p_(*t*, 0) on a scale of *H*^*α*/*2*^ν vs. *t* on a scale of 1*/H*^1/*2*^ν must collapse upon each other^20^; that is why, this procedure is named so. We have found that the collapse (see Fig. 3) takes place at (*α*, ν) = (−0.23, 0.7433) out of any known universality classes. Table 1 in its rows 37–48 contains compounds with similar critical exponents, which, altogether with our Sm_0.55_Sr_0.45_MnO_3_, announce a new universality class for alloys and ternary oxides with a 3*d*-metal cation. As the most of the class is of TTMO, it has been named so. It is intriguing that a high-*T*_c_ superconductor (the row 39) worms its way into that class.

The collapse is observed practically within the all abscissa range, so that no apparent finite-size effects are near *T*_C_. This is no wonder, as, at the mean grain size *L* ≈ 20000 Å finite-size effects could arise only at |*t|* < 0.01 when the correlation radius of magnetic order parameter *ξ* > *L|t|*ν = 652.27 Å, whereas the real *ξ* in Sm_0.55_Sr_0.45_MnO_3_ calculated from its heat capacity^21^ and from its small-angle neutron scattering^22^ is much less. One peculiarity in Fig. 3 attracts attention: the 2.6-T graph at heating superimposes better upon the 3.5-T graph than on the 2.6-T graph at cooling. What, at *T* < *T*_C_ and 2.6 T, the data at cooling is low than that at heating is thought to be caused by embedding lattice distortions inherent to the paramagnetic state into the ferromagnetic state.

Strong magnetic-field effects are emphasized to be distinctive feature of TTMO with the octahedral-centered transition metals: ordering *t*_2g_–orbitals, magnetic field additionally favours to double exchange via the strong spin-orbital interaction of *t*_2g_ and *e*_g_ electrons. Thus, it is natural to suppose that the new TTMO universality class emerges an unknown collective behaviour^1–4,8^. Heisenberg double Hamiltonian^23^ would be useful to take into account lattice distortions during the magnetic transition. In this model, the orbital and spin momenta turn out closely related; and then, varying the spin structure, magnetic field changes the orbital one and so double-exchange interaction. As the result of that, the response of the spin system to magnetic field becomes nonlinear and metamagnetism arises. The interconnection of spins and Jahn-Teller phonons is supposed to account for the inhomogeneities and metamagnetism in the paramagnetic state, resulted in the nonlinear *T*_C_-dependence. Metamagnetic features above *T*_C_ in Sm_0.55_Sr_0.45_MnO_3_ are consistent with the colossal magnetostriction as well as the strong magnetostriction in Jahn-Teller crystals is responsible for their unusual metamagnetism. Moreover, the Heisenberg double model^23^ predicts a tricritical point on the magnetic field – temperature phase diagram.

Some TTMO reveal unconventional criticality and even new universality classes if their magnetic order depends upon a lattice or orbital ordering^24^, hysteresis and tricritical point often observed. Earlier we showed^21^ that the droplet of a nascent phase, a fluctuation of a diameter *ξ*, can appear if the changing of a temperature inside its volume $\frac{4}{3}$π*ξ*^3^ is more of the hysteresis width Δ*T*_C_ = ${T}_{C}^{f-p}-{T}_{C}^{p-f}$. Let us continue the analysis^21^ to deduce a relation between Δ*T*_C_ and *ξ*. Because of colossal striction, a nascent paramagnetic droplet (such a case takes place just below *T*_C_) must work against the elastic force - the surface tension on the spherical boundary of the area *S* = π*ξ*^2^ with the ferromagnetic. According to Hooke’s law, the droplet to grow up from its radius *r* = *ξ*/2 to *r* + *dr* develops the force *F* = π(*E*_fm_ − *E*_pm_)*ξdξ* (*E*_fm_ and *E*_pm_ are the Young moduli in the ferromagnetic and the paramagnetic). Then, the energy $U=\int Fd\xi =\pi ({E}_{{\rm{fm}}}-{E}_{{\rm{pm}}})\int {\xi }^{2}d\xi =\tfrac{\pi }{3}({E}_{{\rm{fm}}}-{E}_{{\rm{pm}}}){\xi }^{3}$, consumed to create the droplet, induces the hysteresis $\Delta {T}_{{\rm{C}}}=\tfrac{\pi }{3}({E}_{{\rm{fm}}}-{E}_{{\rm{pm}}}){\xi }^{3}/{k}_{{\rm{B}}}$. This expression at the zero-field $\Delta {T}_{C}={T}_{C}^{f-p}({\rm{Z}}{\rm{F}}{\rm{H}})-{T}_{C}^{p-f}({\rm{Z}}{\rm{F}}{\rm{C}})$ = 15.3 K and *E*_fm_ and *E*_pm_ from ref. ^25^ gives *ξ* = 8.7 Å in a good agreement with *ξ* yielded from the heat capacity^21^ and small-angle neutron scattering^22,26^. Thus, Coulomb distortions accompanied with colossal striction can restrict *ξ* and so critical behavior.

The tentative TTMO universality class with averaged (*β*, *γ*, *δ*, *α*, *ν*) = (0.45, 1.36, 4.14, −0.25, 0.75) also covers the chemically and physically different compounds: monocrystalline Fe-Pt alloys, amorphous Fe-Mn alloys, the high-*T*_C_ superconductor (Table 1); hence, that class is really universal and its necessity is put forth by us. A peculiarity of the TTMO universality class is that some its members do not obey certain scaling equalities, so that the experimental *δ* and from the Widom equality *δ* = 1 + *γ*/*β* differ. Also *α* of the Rushbrook equality *α* = 2(1 − *β*) − *γ* and of the combination of the Widom and Rushbrook equalities *α* = 2 − *β*(1 + *δ*) are different; that is, the scaling equalities with isothermal *δ* violate. This is because magnetic field directly modifies the lattice behaviour and so changes *δ*. Such behavior is inherent for so-called “Random fixed point” (Table 1). In some compounds, measured *β*, *γ* and *δ* say about their novel criticality, whereas their *α* and *ν*, calculated via scaling relations, belong to ordinary universality classes (Table 1). Hence, *α* and *ν* are more preferable in finding the universality class of a solid.

**Figure 4:**
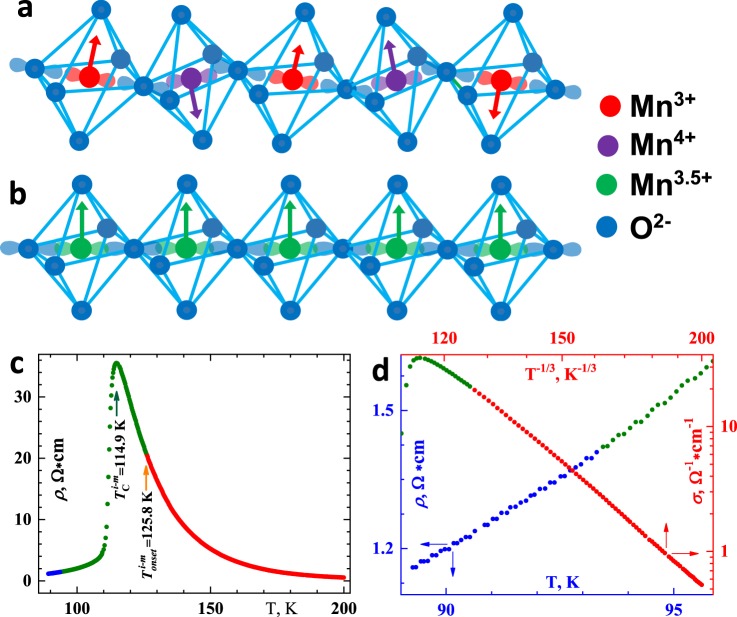
(**a,b)** Pictorial explanation of the coherence of the lattice and magnetic changes in TTMO (Sm_0.55_Sr_0.45_MnO_3_ used as an example). At the high temperatures (800–300 K), ${{\rm{Mn}}}^{4+}{{\rm{O}}}_{6}^{2-}$ and ${{\rm{Mn}}}^{3+}{{\rm{O}}}_{6}^{2-}$ octahedrons are disordered. (**a**) A state below 300 K and up to ${T}_{onset}^{p-f}$. Cooperative Jahn-Teller transition near 300 K leads to ordering the octahedral distortions^22^. Because of the electric charge of Mn^4+^ is more than that of Mn^3+^, Coulomb forces compress ${{\rm{Mn}}}^{4+}{{\rm{O}}}_{6}^{2-}$ more than ${{\rm{Mn}}}^{3+}{{\rm{O}}}_{6}^{2-}$ octahedrons and so form their periodical arrangement. Here is no the overlap of Mn^3+(4+)^*d* and O^2−^*p* orbitals and the magnetic moments of the cations (shown by arrows) disarrange. (**b**) A state below ${T}_{{\rm{C}}}^{p-f}$ enough low to hybridize Mn^3+(4+)^*d* and O^2−^*p* orbitals. The hybridization allows *e*_g_-electron to itinerate from Mn^3+^ via O^2−^ to the nearest Mn^4+^. The quickest (due to the lightest mass of an electron) double exchange of an electron between neighboring the ${{\rm{Mn}}}^{3+}{{\rm{O}}}_{6}^{2-}$ and ${{\rm{Mn}}}^{4+}{{\rm{O}}}_{6}^{2-}$ octahedrons transforms them into the equicharged ${{\rm{Mn}}}^{3.5+}{{\rm{O}}}_{6}^{2-}$, so that the Coulomb distortions annihilate. That corresponds to the onset of the displacive Jahn-Teller transition at ${T}_{onset}^{p-f}$ = 123.3 K obtained from the heat capacity (the insert of Fig. 1). The double exchange of the electron spins forces the magnetic moments of Mn^3.5+^ strictly to arrange and so induces the double-exchange ferromagnetism. In the language of chemistry, the transition to a fractional valence happens and all the physical properties of a solid change - elastic, electric, and magnetic, and etc. (**c,d**) The electrical resistance *ρ* measured by the standard four-point-probe technique and conductance *σ* = 1/*ρ* in Sm_0.55_Sr_0.45_MnO_3_. The pure metallic behavior of the resistance (*ρ* ~ *T*) marked with the blue; the pure bidimensional Mott hopping one (*lgσ* ~ *T* ^−1/3^) with the red. The green segment shows the vicinity of the insulator-to-metal transition (*i-m*) at ${T}_{{\rm{C}}}^{i-m}$ = 114.9 K throughout which the Mott state gradually turns into metallic. ${T}_{{\rm{C}}}^{i-m}$ is slightly, 1.5 K, higher ${T}_{{\rm{C}}}^{p-f}$, and ${T}_{onset}^{i-m}$ is upon 2.5 K above ${T}_{onset}^{p-f}$. Thus, the electrical measurements are more sensitive to detect the beginning of ferromagnetism. (**d**) The conductance up to ${T}_{onset}^{i-m}$ (the red data points) follows the Mott law (see, for example, Mott’s Nobel Lecture) of the hopping conductance in two dimensions lg*σ* ~ *T*^−1/4^. That is ideally supports the reality of the carrier hops along the nonhybridizing *d* and *p* orbitals in the Mn-O planes (see the panel a). The insulator-to-metal transition finishes at 93.4 K (where the green colour changes into blue), and below its temperature (the blue data points) the resistance is linear, that is to say, metal behaviour, which is very good consistent with the conductivity along the overlapping *d* and *p* orbitals (see the panel b) forming the conduction band of the *d*-electrons, takes place. Thus, the resistivity completely supports the explanation of the coherence of the lattice and magnetic changes in TTMO.

**Figure 5:**
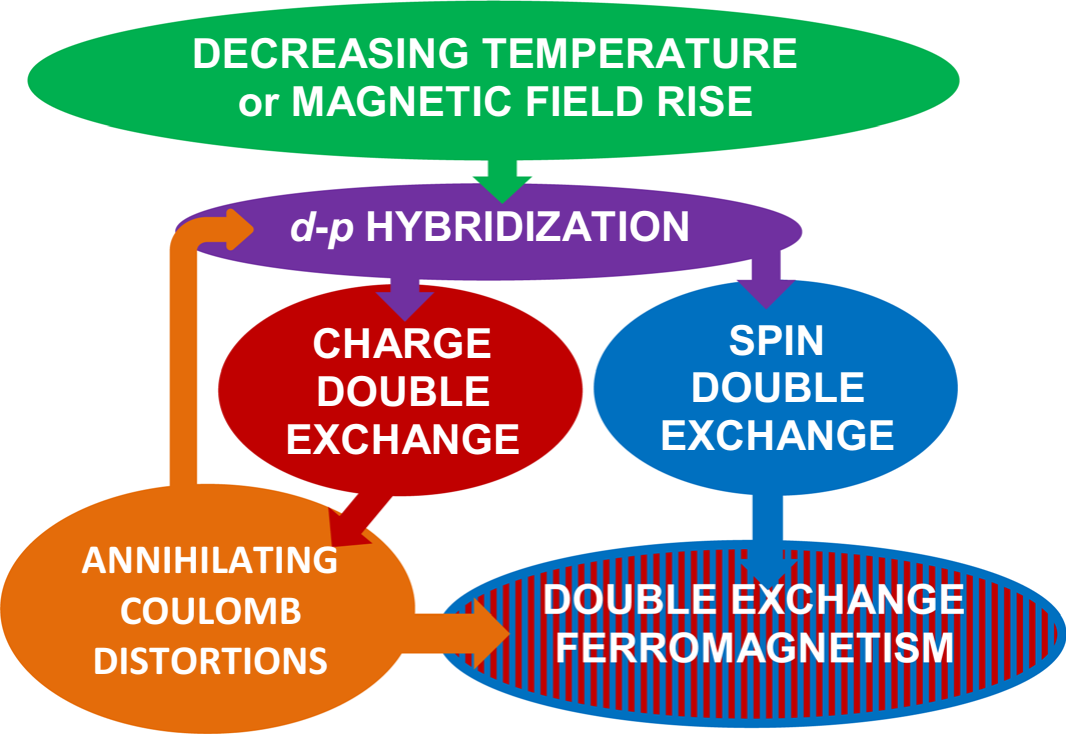
Flowchart understanding the nature of collective behavior in TTMO. When temperature lowers, atoms come nearer to each other; and at last, at *T*_C_, the *d* and *p* orbitals become to touch; also magnetic field, orientating *d*-orbitals and so removing the Coulomb distortions, reaches just the same effect. The overlap allows the electrons of the half-filled *d*-orbitals walk via intermediated *p*-orbitals into the neighboring empty d-orbitals, that is the electrons doubly exchange between neighboring transition-metal cations. At the same time, the double exchange of the electric charges induces the discrete-to-fractional valence transition and so diminishes the Coulomb distortions of the oxygen octahedrons centered with the transition-metal cations. And the double exchange of the electron spins induces double-exchange ferromagnetism. The annihilation of the distortions makes the *d* and *p* orbitals overlap that sufficiently strengthens double-exchange ferromagnetism.

In spite of novel criticality, certain TTMO follow scaling equalities provided their lattice distortions and magnetic order are synchronized by the spin-conservation hops of electrons from one transition-metal cation to the another (Fig. 4a,b). Now, a cause of the coherence of the Coulomb distortion and the double-exchange ferromagnetism pictorially understood, we are able to schematically explain a mechanism of novel criticality in TTMO (see Fig. 5).

The fragmentation of the macroscopic coherent states shown in Fig. 4a,b into mesoscopic clusters with the magnetic dipole-dipole interaction between them instead of double exchange, desynchronizes the lattice and magnetic behaviours, that leads to metamagnetism and so scaling equalities violates (Table 1). That happens when the doping with another transition metal breaks the double-exchange bonds or the doping with another rare earth introduces a disorder in the magnitude of double exchange (see Table 1, rows 4–6). Incidentally, a small drift from the 3*d*-Heisenberg toward the mean-field model in the inhomogeneous SrFe_0.80_Co_0.20_O_3.0_ ferromagnet is supposed to arise from the presence of the dipolar interactions between the Fe^4+^ cations^27^.

Different critical exponents or even distinct universality classes^10^ below and above *T*_C_ are a consequence of hysteresis induced by the Coulomb distortions, which are present above and annihilate below *T*_C_ (Fig. 4a,b). When hysteresis comes to nought at a tricritical point, critical exponents everywhere equal in full accordance with the scaling theory. The influence of the Coulomb lattice distortion on the exchange integral can lead not only to novel criticality but also to metamagnetism which violates the scaling equalities between isothermal and isomagnetic exponents (the short-range antiferromagneic correlations in Cu_1−x_NMn_3+x_ is a classic feature of metamagnetism^9^).

The lattice, Mott, magnetic and ferroelectric transitions inherent TTMO give diversity of their interdependence: the critical temperatures nonlinear and very-sensitive to magnetic and electric fields and pressure; hysteresis and so tricritical points; and, finally, novel criticality which is the culmination of the all. So, discovering the nature of the novel criticality is very important for understanding of amazing properties of TTMO in tailoring principal-new spintronic devices on the base of the film heterostructures^27–30^ of the alternated layers of a TTMO with the ferroelectric, insulator, semiconductor, semimetal, or superconductor.

## Conclusions

In summary, we studied the magnetic heat-capacity scaling of Sm_0.55_Sr_0.45_MnO_3_ manganite using heat-capacity measurements in magnetic fields up to 4 T. We found that the critical exponent of the heat capacity *α* and the correlation radius ν were out of any known universality classes, which was attributable to the existence of Coulomb distortions, as demonstrated by the neutron-diffraction result and as supported with the electrical resistance and the electric conductance. The disappearance of Coulomb distortions near *T*_C_ leads to the colossal striction and the elastic energy of the lattice lowers, so that not only the spin of an electron, but also its electric charge makes ferromagnetism favourable. That nonlinear effect strengthens the critical behaviour and critical exponents come off any known universality classes. The long-range coherence of the Coulomb distortions and ferromagnetism remains scaling equalities, however, its violation breaks off the scaling equalities between isothermal and isomagnetic exponents. The present study announces the new universality class for TTMO and other system with the similar critical exponents (see Table 1). As the similar critical phenomena take place in the diversity of compounds thoroughly reviewed in Table 1, the proposed mechanism of novel criticality would be fruitful in interpreting their unconventional critical properties. Hence, the novelty of the Report is in presenting the successful heat-capacity scaling the manganite; the explanation of the origin of the novel criticality in TTMO; and the new universality class including compounds (Table 1, rows 37–48) with unordinary critical exponents which nature was unknown before. That open new perspectives for studying the criticality in various systems to advance in understanding machanisms of phase transitions in them. Heat capacity is a universal property of the matter, therefore its scaling analysis is suitable for any solids, including those in which the scaling of electrical resistivity (an insulator) or of magnetization and/or magnetic susceptibility (a paramagnetic and an antiferromagnetic) is practically impossible. The heat-capacity scaling, for example, gives possibility to carry out the transition of the paramagnetic Mott insulator k-(BEDT-TTF)_2_Cu[N(CN)_2_]Cl into antiferromagnetic insulator. Besides that, it would be very interest to study in the same substance a scaling of the transition from antiferromagnetic insulator to unconventional superconductivity with pressure to reveal whether the new universality class found by us realizes; and if so, we would have an evidence of the synergic effect of the lattice and magnetic modifications on the superconductivity. The high compressibility of lattice and hysteresis^4^ in k-(BEDT-TTF)_2_Cu[N(CN)_2_]Cl are in favour of this. Our findings are thought to be useful, for example, in interpreting the intriguing coincidence of the electrical, structure and magnetic transitions in the Mn-doped chalcopyrites^31^ and the Dirac semimetal^32^.

## Methods

The Sm_0.55_Sr_0.45_MnO_3_ manganite was prepared by the chemical homogenizing method^33,34^ from aqueous solutions of Sm, Sr, and Mn nitrates with a total concentration of 1 mol/l. The residue left after the burning of these filters was calcined at 973 K, pressed into pellets, and sintered at 1473 K for 12 h. The phase composition of the ceramics and the lattice parameters were characterized by X-ray diffraction (Fig. 2a) with a Siemens D5000 diffractometer. The obtained ceramics were found to be a single-phase perovskite with orthorhombic structure (*P*_nma_ group) and the lattice parameters: *a* = 5.424(1) Å, *b* = 7.678(2) Å, and *c* = 5.434(2) Å. The value of the orthorhombicity parameter, 0.2%, suggests closeness to the cubic structure. The ratio *a* < *b*$\sqrt{2}$ < *c* is characteristic of orthorhombic manganites with a tolerance factor of ≈0.92. The sample density is 5.16 g/cm^3^.

**Figure 6:**
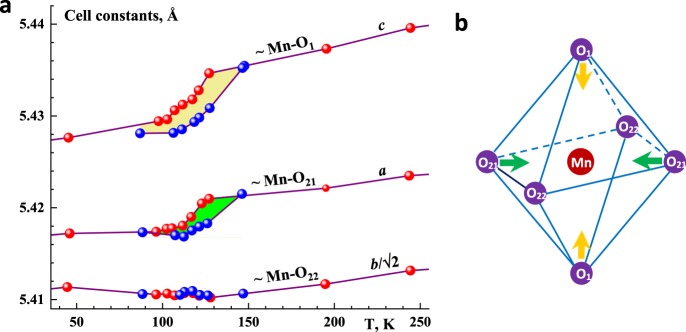
(**a**) Temperature dependences of unit cell parameters in ^152^Sm_0.55_Sr_0.45_MnO_3_ derived from G4.2 measurements. Red balls refer to the heating mode, and the blues to the cooling mode. The hysteresis of the lattice constants *a* and *c*, shown with the yellow and green fillings, are consistent with the one in the heat-capacity. (**b**) Octahedron MnO_6_ with the designations of the apical and basal oxygen atoms. The separations of Mn-O_1_, Mn-O_21_ and Mn-O_22_ are 1.953(2), 1.955(6) and 1.931(7) Å before the transition into ferromagnetic state (at 150 K), and 1.947(3), 1.945(8) and 1.937(6) after (at 100 K). The relative change of the octahedrons volume V = $\frac{4}{3}$[Mn-O_1_][Mn-O_21_][Mn-O_22_] through the magnetic transition is 0.51%. The Jahn-Teller striction modes of the octahedron (marked with green and yellowish arrows) are proportional to the distortions in *a* and *c* lattice constants near the displacive Jahn-Teller transition, highlighted with the same colours on the (a) panel. In the Report, the type of Coulomb lattice distortion in Sm_0.55_Sr_0.45_MnO_3_ is not specified as many kinds of it, for example, metamagnetism^9^, lattice changings^2^, and ferroelectric deformation side by side with Jahn-Teller effect cause unordinary criticality.

^152^Sm_0.55_Sr_0.45_MnO_3_ neutron-diffraction patterns^35–37^, measured at different temperatures and a neutron wavelength 2.343 Å on a G4.2 high-resolution neutron powder diffractometer at the neutron guide room of an ORPHEUS reactor (LLB, Saclay, France), show no additional peaks attributed to antiferromagnetism below ${T}_{C}^{p-f}({\rm{ZFC}})$ and no phase separation (Fig. 2b). Sm_0.55_Sr_0.45_MnO_3_ suffers a phase transition into a homogeneous ferromagnetic metallic-like state^21,22^ with the magnetic moment *M* = 3.36(5) *μ*_B_ per a Mn atom upon saturation at *T* = 4 K that corresponds to a complete ferromagnet order without indications of antiferromagnetism. Thus, Sm_0.55_Sr_0.45_MnO_3_ possesses the maximum ferromagnetic fraction that allows us to choose it to carry out the renewed scaling procedure^12^. The neutron-diffraction data near ${T}_{C}^{p-f}({\rm{ZFC}})$ indicate an abrupt decrease in the cell volume (*ΔV*_C_/*V*_C_ ≈ 0.1%) upon transition into the ferromagnetic state, associated with a displacive Jahn-Teller transition, that is, the annihilation of Coulomb distortions, the space group, *P*_nma_, remaining over the all temperatures. The lattice parameters changed in a specific manner: the rhombic base of the unit cell contracted sharply (the temperature dependences of the parameters *a* and *c* exhibited jumps), whereas the parameter *b* changed only slightly (Fig. 6a). The orthorhombic distortion *δ* = (*a* − *c*)/(*a* + *c*) ≈ 0.15% which is comparatively large. The Curie point is attended by jump-wise decreasing the Coulomb distortions of the MnO_6_-octahedrons. The neutron diffraction patterns have given detailed data on the Coulomb distortions of the MnO_6_-octahedrons (Fig. 6b). The angle Mn-$\widehat{{{\rm{O}}}_{21}}$-Mn, an angle between the adjacent octahedrons, at room temperature ≈159° but ≈161° near ${T}_{C}^{p-f}({\rm{ZFC}})$. The Coulomb distortion parameter ($\frac{1}{3}$Σ[(Mn-O_*i*_) − 〈Mn-O〉]^2^)^1/2^_*i* = 1, 21 and 22_ ≈ 0.01 Å at 150 K and ≈0.004 Å at 100 K. So, the changings of the interatomic distances interior the octahedron and the angle between adjacent octahedron indicate noticeable Coulomb distortions above *T*_C_ and their significant reduction below *T*_C_, which obviously affect double exchange and, hence, the critical behavior.

**Figure 7:**
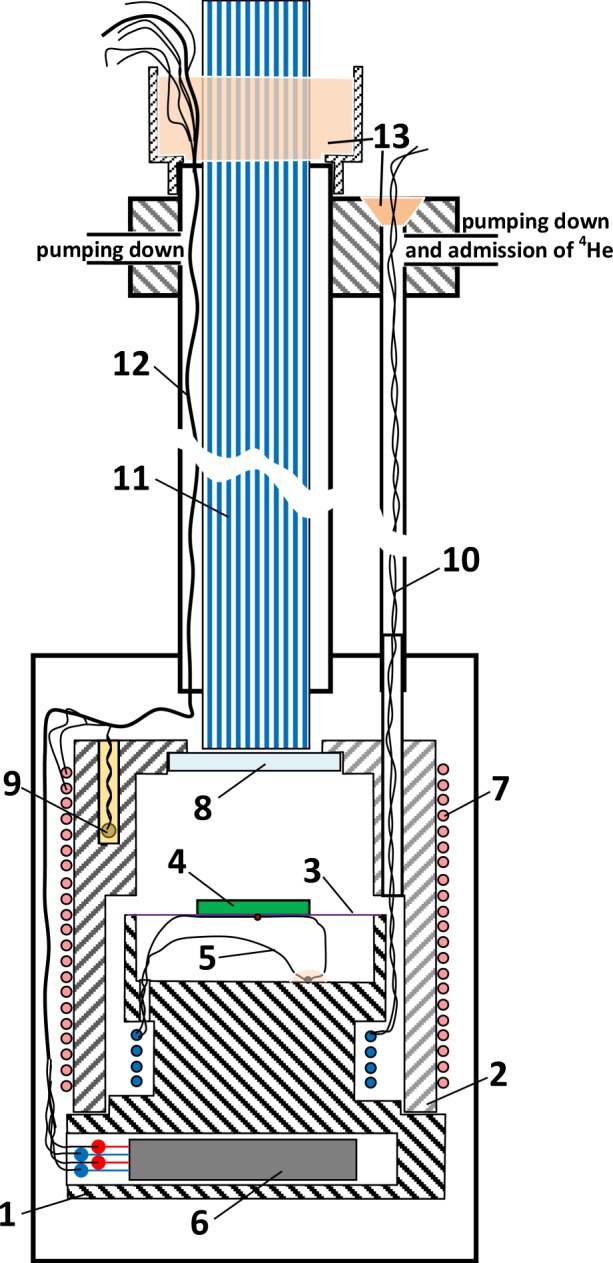
The heat-capacity measuring apparatus has a base 1, a cover 2 and two glass fibres 3 on which there is a sample 4 onto which a thermocouple 5 measuring its temperature oscillations T_*f*_ is glued. A carbon resistance thermometer 6 is placed in a hole in the base. A heater 7 is wound on the outer surface of the cover. A calorimeter thermocouple 9 measuring the average temperature of the thermal bath T_bath_ is buried on one side of the top cover and on the other side of the cover there is a calorimeter holder tube 10 which also used to pass the thermocouple lead wires and pumping down the thermal bath and for admission the gaseous ^4^He. Above the window 8 (glued to the inside of the cover with Araldite to provide a hermetic seal down to the lowest temperatures), there is a light guide 11 which runs to the calorimeter along the tube 12, used also for pumping down and to pass the lead wires of the T_bath_-thermocouple, the carbon resistance thermometer and the heater. The light guide and wires is sealed with the half-and-half mix of beeswax and colophony 13.

The heat capacity *C*_p_ was measured using an apparatus (Fig. 7) by an ac-calorimetry^38^. One advantage of the method is the smallest temperature gradient across a sample (<10 mK), which is especially important in studying the critical phenomena near *T*_C_. The gradients permit measurements with sensitivity less than 0.01 K or 10^−5^ of the reduced temperature *|T*/*T*_C_ − 1|. According to the ac-calorimetry, absorbing a light energy per a second *dQ* from an tungsten lamp, modulated with a frequency *f*, leads to the sample temperature oscillations *T*_*f*_ with the same frequency and increases an average sample temperature of the *T* with respect to a thermal-bath temperature *T*_bath_ on *T*_dc_, i.e. *T* = *T*_bath_ + *T*_dc_. By measuring *T*_dc_ and *T*_*f*_ with known the temperature dependence of heat conduction, *g*(*T*), of gaseous ^4^He filling the thermal bath, and a distance between the sample and the thermal bath, *d*, the absolute value of *C*_p_ = *dg*(*T*)*T*_dc_/*T*_*f*_ (*T*_dc_ used not to exceed 1.5 K). To reach the quickest response to a temperature of the sample, the 25-μm chromel-constantan thermocouple, spot-welded from the flattened ends of the wires, was glued to the backside of the sample with the BF-2 phenol polyvinyl-acetal glue (the analog of the GE-7031 varnish). The sample is a 0.27-mm thickness platelet with the area of 3*3 mm^2^ and weight <30 mg. The “cold junction” of the thermocouple is mounted on the thermal bath to simultaneously detect *T*_*f*_ and *T*_dc_. The front side of the sample was periodically lit by a tungsten lamp with a mechanical chopper. The used frequency *f* = 2 Hz adopts the ac-calorimetry criteria^38^ and allows us to set the 10-s time constant on a lock-in amplifier to maximally avoid the electromagnetic noise. The 0.1-mm copper-constantan thermocouple with one junction glued to the thermal bath and another placed in the ice bath detected *T*_bath_. The absolute error 0.8% was estimated as the ratio of the heat capacities of the thermocouple and the adjacent sample volume bounded by the thermal diffusivity length $l=\sqrt{\eta /\pi f}$, a distance on which the absorbed heat propagates for a period (*η* is the thermal diffusivity).

## References

[1] Griffin SM (2012). Scaling behavior and beyond equilibrium in the hexagonal manganites. Phys. Rev. X.

[2] Omerzu A, Tokumoto M, Tadic B, Mihailovic D (2001). Critical exponents at the ferromagnetic transition in tetrakis(dimethylamino)ethylene-C_60_ (TDAE-C_60_). Phys. Rev. Lett..

[3] Limelette P (2003). Universality and critical behavior at the Mott Transition. Science.

[4] Kagawa F, Miyagawa K, Kanoda K (2005). Unconventional critical behaviour in a quasi-two dimensional organic conductor. Nature.

[5] Jiang WJ, Zhou XZ, Williams G, Mukovskii Y, Glazyrin K (2007). Is a griffiths phase a prerequisite for colossal magnetoresistance?. Phys. Rev. Lett..

[6] Zhou JS (2008). Critical behavior of the ferromagnetic perovskite BaRuO_3_. Phys. Rev. Lett..

[7] Sarkar P (2009). Pressure induced critical behavior of ferromagnetic phase transition in Sm-Nd-Sr manganites. Phys. Rev. Lett..

[8] Tateiwa N, Haga Y, Matsuda TD, Yamamoto E, Fisk Z (2014). Unconventional critical scaling of magnetization in ferromagnetic uranium superconductors UGe_2_ and URhGe. Phys. Rev. B.

[9] Lin J (2015). Unusual ferromagnetic critical behavior owing to short-range antiferromagnetic correlations in antiperovskite Cu_1-x_NMn_3+x_ (0.1 ≤ × ≤ 0.4). Sci. Rep..

[10] Ginting D (2015). Second order magnetic phase transition and scaling analysis in iron doped manganite La_0.7_Ca_0.3_Mn_1-x_Fe_x_MnO_3_ compounds. JMMM.

[11] Lin P, Chun SH, Salamon MB, Tomioka Y, Tokura Y (2000). Magnetic heat capacity in lanthanum manganite single crystals. J. Appl. Phys..

[12] Abdulvagidov SB, Djabrailov SZ (2017). Improved scaling of the magnetic heat capacity in La_0.85_Ag_0.15_MnO_3_ manganite. JETP Lett..

[13] Anderson PW, Hasegawa H (1955). Considerations on Double Exchange. Phys. Rev..

[14] Asamitsu A, Moritomo Y, Tomioka Y, Arima T, Tokura Y (1995). A structural phase transition induced by external magnetic field. Nature.

[15] Asamitsu A, Tomioka Y, Kuwahara H, Tokura Y (1997). Current switching of resistive states in magnetoresistive manganites. Nature.

[16] Myers EB, Ralph DC, Katine JA, Louie RN, Buhrman RA (1999). Current-induced switching of domains in magnetic multilayer devices. Science.

[17] Thomas L (2007). Resonant amplification of magnetic domain-wall motion by a train of current pulses. Science.

[18] Choi S (2017). Switching magnetism and superconductivity with spin-polarized current in iron-based superconductor. Phys. Rev. Lett..

[19] Oleaga A, Salazar A, Prabhakaran D, Cheng J-G, Zhou JS (2012). Critical behavior of the paramagnetic to antiferromagnetic transition in orthorhombic and hexagonal phases of *R*MnO_3_ (*R* = Sm, Tb, Dy, Ho, Er, Tm, Yb, Lu, Y). Phys. Rev. B.

[20] Stainley, H.E. *Introduction to Phase Transition and Critical Phenomena*, Oxford University Press. London, (1971).

[21] Abdulvagidov SB, Kamilov IK, Aliev AM, Batdalov AB (2003). Heat capacity and electric resistance of Sm_0.55_Sr_0.45_MnO_3_ manganite near *T*_C_ in a magnetic field of up to 26 kOe: fluctuation effects and colossal magnetoresistance development scenario. JETP.

[22] De Teresa JM (2002). Magnetic versus orbital polarons in colossal magnetoresistance manganites. Phys. Rev. B.

[23] Kugel KI, Khomskii DI (1976). Heisenberg model in a magnetic field, and metamagnetism of Jahn-Teller systems. JETP Lett..

[24] Rößler S (2011). Ferromagnetic transition and specific heat of Pr_0.6_Sr_0.4_MnO_3_. Phys. Rev. B.

[25] Troyanchuk IO, Khomchenko VA, Tovar M, Szymczak H, Bärner K (2004). Antiferromagnet-ferromagnet and structural phase transitions in La_0.88_MnO_*x*_manganites. Phys. Rev. B.

[26] De Teresa JM (1997). Evidence for magnetic polarons in the magnetoresistive perovskites. Nature.

[27] Lago J (2011). Critical behavior in the inhomogeneous ferromagnet SrFe_0.80_Co_0.20_O_3.0_. Phys. Rev. B.

[28] Hepting M (2018). Complex magnetic order in nickelate slabs. Nat. Phys..

[29] Fei Z (2018). Ferroelectric switching of a two-dimensional metal. Nature.

[30] Deng Y (2018). Gate-tunable room-temperature ferromagnetism in two-dimensional Fe_3_GeTe_2_. Nature.

[31] Arslanov T (2015). Pressure control of magnetic clusters in strongly inhomogeneous ferromagnetic chalcopyrites. Sci. Rep..

[32] Li H (2015). Negative magnetoresistance in Dirac semimetal Cd_3_As_2_. Nat. Commun..

[33] Melnikov OV (2006). Electrical and magnetic properties of La_1-x_Ag_y_MnO_3_ recrystalized ceramics. Funct. Mater..

[34] Gorbenko OY (2005). Solid solutions La_1-x_Ag_y_MnO_3+δ_: evidence for silver doping, structure and properties. Mater. Sci. Eng. B.

[35] Kurbakov AI, Trunov VA, Andre G (2004). Study of effect isotopic substitution ^16^O→^18^O in Sm_1-x_Sr_x_MnO_3_–type (x = 0.45 and 0.50) manganites by powder neutron diffraction. Crystallography Reports.

[36] Lazuta AV (2003). Magic hole doped composition of ^152^Sm_1−x_Sr_x_MnO_3_manganite: crystal structure and unusual magnetic properties in paramagnetic phase at x = 0.45. JMMM.

[37] Kurbakov AI (2010). Electronic, structural and magnetic phase diagram of Sm_1-x_Sr_x_MnO_3_ manganites. JMMM.

[38] Sullivan P, Seidel G (1968). Steady-State, ac-Temperature Calorimetry. Phys. Rev..

[39] Ginting D, Nanto D, Zhang YD, Yu SC, Phan TL (2013). Influences of Ni-doping on critical behaviors of La_0.7_Sr_0.3_Mn_1−x_Ni_x_O_3_. Physica B.

[40] Khan N (2010). Critical behavior in single-crystalline La_0.67_Sr_0.33_CoO_3_. Phys. Rev. B.

[41] Yamada K, Ishikawa Y, Endoh Y, Masumoto T (1975). The magnetic phase transition of an amorphous Fe, P, C and its alloys containing Ni and Cr. Solid State Commun..

[42] Kouvel JS, Comly JB (1968). Magnetic equation of state for nickel near its Curie point. Phys. Rev. Lett..

[43] Poon SJ, Durand J (1977). Critical phenomena and magnetic properties of an amorphous ferromagnet: Gadolinium-gold. Phys. Rev. B.

[44] Deschizeaux MN, Develey G (1971). Magnetic equation of state of gadolinium near the Curie point. J. Phys. (Paris).

[45] Salamon MB, Chun SH (2003). Griffiths singularities and magnetoresistive manganites. Phys. Rev. B.

[46] Phan TL (2017). Tricritical behavior and Griffith phase in La_1-x_Ca_x_MnO_3_ under high applied fields. JMMM.

[47] Collins MF, Minkiewicz VJ, Nathans R, Passell L, Shirane G (1969). Critical and spin-wave scattering of neutrons from iron. Phys. Rev..

[48] Yiang J, Lee YP, Li Y (2007). Critical behavior of the electron-doped manganite La_0.9_Te_0.1_MnO_3_. Phys. Rev. B.

[49] Fan J (2010). Critical properties of perovskite manganite La_0.1_Nd_0.6_Sr_0.3_MnO_3_. Phys. Rev. B.

[50] Huang, K. *Statistical Mechanics*, 2nd ed., Wiley, New-York (1987).

[51] Robinson DS, Salamon MB (1982). Universality, Tricriticality, and the Potts transition in first-stage lithium-intercalated graphite. Phys. Rev. Lett..

[52] Boxberg O, Westerholt K (1994). Critical exponents at the ferromagnetic phase transition of Fe_100-x_Pt_x_ single crystals. Phys. Rev. B.

[53] Yeshurun Y, Salamon MB, Rao KV, Chen HS (1980). Spin-glass-ferromagnetic critical line in amorphous Fe-Mn alloys. Phys. Rev. Lett..

[54] Salamon MB (1988). Effect of magnetic fields on the specific heat of a YBa_2_Cu_3_O_7−δ_ single crystal near *T*_c_. Phys. Rev. B.

[55] Jiang W, Zhou XZ, Williams G, Mukovskii Y, Glazyrin K (2008). Griffiths phase and critical behavior in single-crystal La_0.7_Ba_0.3_MnO_3_: Phase diagram for La_1−*x*_Ba_*x*_MnO_3_(*x* ≤ 0.33). Phys. Rev. B.

[56] Mira J (1999). Critical exponents of the ferromagnetic-paramagnetic phase transition of La_1*-*x_Sr_x_CoO_3_ (0.20< × <0.30). Phys. Rev. B.

[57] Figueroa E, Lundgren L, Beckman O, Bhagat SM (1976). The anomalous magnetisation of amorphous metglas 2826-A. Solid State Commun..

[58] Kida T (2008). Unconventional critical behavior in the weak ferromagnet BaIrO_3_. European Phys. Lett..

